# O6-methylguanine-DNA methyltransferase in pretreatment tumour biopsies as a predictor of response to temozolomide in melanoma.

**DOI:** 10.1038/bjc.1998.654

**Published:** 1998-11

**Authors:** M. R. Middleton, J. M. Lunn, C. Morris, G. Rustin, S. R. Wedge, M. H. Brampton, M. J. Lind, S. M. Lee, D. R. Newell, N. M. Bleehen, E. S. Newlands, A. H. Calvert, G. P. Margison, N. Thatcher

**Affiliations:** Department of Medical Oncology, Christie Hospital NHS Trust, Manchester, UK.

## Abstract

Resistance of tumour cells to methylating and monochloroethylating agents in vitro and in vivo has been linked to levels of the DNA repair protein O6-methylguanine-DNA methyltransferase (MGMT). In a clinical trial of temozolomide in advanced malignant melanoma, the relationship between pretreatment MGMT levels in biopsies of cutaneous tumours and involved lymph nodes and clinical response to the drug has been studied. Among 50 evaluable patients, there were three complete responses (CR), four partial responses (PR), six with stable disease (SD) and 37 with progressive disease (PD), with an overall response rate of 14%. In 33 patients in whom MGMT level and clinical response could be evaluated, the tumour MGMT levels (fmol mg(-1) protein) were: CR, 158 +/- 119; PR, 607 +/- 481; NC, 171 +/- 101; PD, 185 +/- 42.3. Thus, measurements of pretreatment levels of MGMT in melanoma did not predict for response to temozolomide.


British Journal of Cancer (1998) 78(9), 1199–1202
© 1998 Cancer Research Campaign

### $O^6$-Methylguanine-DNA methyltransferase in pretreatment tumour biopsies as a predictor of response to temozolomide in melanoma

**MR Middleton[1], JM Lunn[2], C Morris[1], G Rustin[3], SR Wedge[4], MH Brampton[5], MJ Lind[2], SM Lee[1], DR Newell[2], NM Bleehen[6], ES Newlands[4], AH Calvert[2], GP Margison[1] and N Thatcher[1]**

[1]Department of Medical Oncology, Christie Hospital NHS Trust, Wilmslow Road, Manchester M20 9BX, UK; [2]Cancer Research Unit, The Medical School, University of Newcastle upon Tyne, Newcastle upon Tyne NE2 4HH, UK; [3]Cancer Treatment Centre, Mount Vernon Hospital, Rickmansworth Road, Northwood, Middlesex HA6 2RN, UK; [4]Department of Medical Oncology, Charing Cross Hospital, Fulham Palace Road, London W6 8RF, UK; [5]32 Howe End, Kirbymoorside, York YO6, 6BD, UK; [6]Department of Clinical Oncology and Radiotherapy, Addenbrooke's Hospital, Hills Road, Cambridge CB2 2QQ, UK; on behalf of the Cancer Research Campaign Phase I/II Committee

**Summary** Resistance of tumour cells to methylating and monochloroethylating agents in vitro and in vivo has been linked to levels of the DNA repair protein $O^6$-methylguanine-DNA methyltransferase (MGMT). In a clinical trial of temozolomide in advanced malignant melanoma, the relationship between pretreatment MGMT levels in biopsies of cutaneous tumours and involved lymph nodes and clinical response to the drug has been studied. Among 50 evaluable patients, there were three complete responses (CR), four partial responses (PR), six with stable disease (SD) and 37 with progressive disease (PD), with an overall response rate of 14%. In 33 patients in whom MGMT level and clinical response could be evaluated, the tumour MGMT levels (fmol $mg^{-1}$ protein) were: CR, 158 ± 119; PR, 607 ± 481; NC, 171 ± 101; PD, 185 ± 42.3. Thus, measurements of pretreatment levels of MGMT in melanoma did not predict for response to temozolomide.

**Keywords:** $O^6$-methylguanine-DNA methyltransferase; temozolomide; melanoma; response

Less than one-third of patients with metastatic melanoma respond to the best available single agent, dacarbazine (Lee et al, 1995). Despite the development of combination regimens, the median progression-free interval remains short and median survival is only 6 months. It would be helpful to identify those patients unlikely to respond to alkylating agents to spare them potentially toxic therapy and allow them to be considered for alternative approaches to treatment.

The Cancer Research Campaign (CRC) has identified a new alkylating agent, temozolomide (Newlands et al, 1997), which has activity against melanoma comparable to that of dacarbazine (Bleehen et al, 1995). The agent's mechanism of action depends upon the methylation of guanine bases in DNA at the $O^6$ position (Margison and O'Connor, 1990). Unrepaired, the lesion can result in chain termination, or initiate ineffective cycles of mismatch repair leading to strand-break formation (Karran and Bignami, 1992; Griffin et al, 1994; Voigt and Topal, 1995). However, the $O^6$-methyl adduct can be removed by the protein $O^6$-methylguanine-DNA methyltransferase (MGMT) in a stoichiometric auto-inactivating reaction. There is evidence that MGMT expression is a major determinant of cellular susceptibility to methylating agent chemotherapy; tumour cell lines or xenografts with high levels of protein expression are more resistant to temozolomide and related agents than those which are deficient in MGMT (Yarosh et al, 1986; Catapano et al, 1987; D'Incalci et al, 1988; Margison and O'Connor, 1990; Pegg, 1990).

Clinical responses to methylating, or other $O^6$-alkylating, therapies in relation to MGMT expression have been less widely studied. Recently, an inverse correlation between clinical response and tumour cell MGMT concentration has been reported in leukaemia patients treated with dacarbazine (Franchi et al, 1992), and glioma patients treated with a chloroethylnitrosourea (Yanagisawa et al, 1996). However, in both studies numbers were small, and the distinction between high and low MGMT levels of expression was made retrospectively.

We have examined prospectively the relationship between tumour MGMT concentration, measured in biopsies of cutaneous melanoma or lymph node metastases, and response to treatment with temozolomide in patients with advanced malignant melanoma. Comparison between pretreatment MGMT levels in peripheral blood mononuclear cells and tumour biopsies has also been made in a subset of patients.

### MATERIALS AND METHODS

### Patient selection, treatment and evaluation

Patients with progressive advanced malignant melanoma were eligible for the study. The inclusion criteria were a lesion accessible for biopsy, measurable disease and adequate organ function. A WHO performance status of 3 or less was required, and previous chemotherapy and/or radiotherapy were permitted provided that 4 weeks had elapsed from the last treatment and any toxicity had resolved. Sixty-one patients were registered for the trial at four centres between July 1994 and September 1996, of whom three

*Received 15 December 1997*

*Revised 3 April 1998*

*Accepted 7 April 1998*

*Correspondence to:* MR Middleton, Department of Medical Oncology, Christie Hospital NHS Trust, Wilmslow Road, Manchester M20 9BX, UK

**Table 1** Characteristics of evaluable patients

| | Number | (Percentage) |
|---|---|---|
| Total number of patients | 56 | |
| Men | 26 | (46) |
| Women | 30 | (54) |
| WHO performance status | | |
| 0 | 17 | (30) |
| 1 | 33 | (59) |
| 2 | 5 | (9) |
| 3 | 1 | (2) |
| Disease sites at entry | | |
| Soft tissue | 18 | (32) |
| Visceral (not CNS) | 32 | (54) |
| CNS | 8 | (14) |
| Prior treatment | | |
| Surgery | 56 | (100) |
| Radiotherapy | 12 | (21) |
| Chemotherapy[a] | 11 | (20) |
| Biotherapy | 2 | (4) |

[a]Including chemobiotherapy; nine of the patients had received DTIC-based regimens.

**Table 2** Characteristics of responding patients

| Age/gender | Disease sites | Prior therapy | Response duration (days) |
|---|---|---|---|
| Complete responders | | | |
| 60F | Soft tissue | Surgery only | 944 (ongoing) |
| 58M | Soft tissue | Surgery only | 145 |
| 63F | Soft tissue | Limb perfusion[a] | 629 (ongoing) |
| Partial responders | | | |
| 32F | Visceral | Surgery only | 207 |
| 48M | Visceral | Surgery only | 140 |
| 50F | Visceral | Surgery only | 140 |
| 58F | Soft tissue | Surgery only | 194 |

[a]With 5-fluorouracil.

**Table 3** MGMT levels in each category of response

| | Number of patients | MGMT (fmol mg$^{-1}$ protein) ± s.e. |
|---|---|---|
| CR | 3 | 158 ± 119 |
| PR | 3 | 607 ± 481 |
| SD | 3 | 171 ± 101 |
| PD | 24 | 185 ± 42.3 |

proved ineligible (one with no measurable disease, one with inadequate hepatic function and one with inadequate renal function) and two were lost to follow up. Characteristics of the remaining 56 patients are shown in Table 1. On the first treatment cycle, temozolomide (supplied by the CRC Drug Formulation Unit, University of Strathclyde, UK) was given at 150 mg m$^{-2}$ daily by mouth for 5 days. If myelotoxicity was grade 0 or 1, the dose was increased to 200 mg m$^{-2}$ day$^{-1}$ for subsequent cycles. Cycles were repeated every 28 days.

Responses were determined according to WHO criteria (1979). Patients had to receive at least two cycles of treatment so that response could be compared with the tumour biopsy MGMT level.

**Figure 1** Pretreatment tumour biopsy MGMT concentration in comparison with clinical response

Those who were unable to complete two cycles of treatment were assessed for toxicity, according to CTC criteria (Miller et al, 1981), and response only. The trial was approved by the appropriate local ethical review committees.

### Tumour biopsy MGMT determination

Biopsies of cutaneous melanoma or lymph node metastases were taken before the treatment started, and extracts prepared for measurement of MGMT expression. Levels in tumour biopsy samples were determined in three of the four participating centres, according to the methods of Lee et al (1991) or Major et al (1991). These methods quantify MGMT activity by measuring the transfer of tritiated methyl groups from DNA to the protein fraction, containing MGMT, in cell extracts. A number of samples were analysed at all three laboratories to ensure consistency of measurement. In a subset of patients, peripheral blood was collected immediately before treatment, mononuclear cells separated by Ficoll–Hypaque density centrifugation and analysed as above.

### RESULTS

Treatment was well tolerated; only one patient was withdrawn from the study because of drug toxicity, suffering prolonged grade IV thrombocytopenia. Lymphocytopenia was common (occurring in 85% of patients), but there were only eight (4% of cycles) and nine (5%) reports of grade 3 or higher neutropenia and thrombocytopenia, respectively, in 189 cycles of treatment. The most frequent non-haematological toxicities were nausea (in 60% of patients), vomiting (45%), constipation (43%), diarrhoea (21%) and stomatitis (12%). Five patients could not be assessed because they died before completing a cycle of treatment. All these deaths were due to progressive malignant melanoma except one. The exception was a patient who died of pneumonia 21 days into the first cycle of treatment, which was not obviously related to temozolomide use.

Clinical response was not evaluable in one patient. In the remaining 50 patients, there were three (6%) complete responses (CR), four (8%) partial responses (PR), six (12%) patients with stable disease (SD) and 37 (74%) with progressive disease (PD), giving an overall response rate of 14%. The characteristics of the

*© Cancer Research Campaign 1998*

responding patients are summarized in Table 2. Median time to progression for all evaluable patients was 57.5 days and median survival 159.5 days.

In 17 of the 50 evaluable patients, response could not be assessed against tumour biopsy MGMT level: seven had early progression and ten had biopsies which were inadequate for assessing the protein level. Results from the 33 patients in whom both response and MGMT expression could be examined are presented in Table 3 and Figure 1. There was no significant difference in pretreatment MGMT levels between those who responded to temozolomide and those who did not ($P = 0.95$: Mann–Whitney test). MGMT levels did not correlate with an individual's time to progression nor overall survival. In ten patients, there was no linear ($r = 0.045$, $P$ = NS) or rank ($r = 0.097$, $P = 0.40$) relationship between pretreatment peripheral blood mononuclear cell and tumour biopsy MGMT levels.

### DISCUSSION

The overall response rate of 14% observed in the present study is lower than that seen in the earlier CRC phase II study of temozolomide in advanced malignant melanoma (Bleehen et al, 1995). However, patients in the earlier study were chemotherapy naive and had a better median performance status at the start of temozolomide therapy, and only one patient had CNS disease (compared with eight in this study). No patient in the current trial who had received previous systemic chemotherapy responded to temozolomide. The majority of the regimens given before temozolomide included dacarbazine, which shares the active intermediate 5-(3-methyl-1-triazenyl)imidazole-4-carboxamide with temozolomide, so that cross-resistance is not unexpected. However, there was no difference in the mean MGMT levels of patients who had received prior chemotherapy and those who had not (data not shown). The toxicities seen in this study are similar to those in the previous melanoma trial and other phase II studies (Bleehen et al, 1995; Bower et al, 1997).

These results show no relationship between averaged tumour MGMT activity and response to temozolomide chemotherapy. Although there are a number of other variables to take into account (such as drug absorption, metabolism and penetration to tumour sites), this is surprising in the face of the large body of preclinical in vitro and xenograft evidence for such a correlation. However, the measure of tumour MGMT used in this investigation may not have been representative of the tumour as a whole. Thus, it may not be reasonable to view the tumour as a single entity with regard to MGMT activity, given the heterogeneity of expression found in immunohistochemical studies (Lee et al, 1992) or the different levels of expression found in multiple skin metastases biopsied in the same patient (Eghyazi et al, 1995). Although MGMT was measured in samples pared of non-tumour tissue visible to the naked eye, the activity recorded represents the mean for all the cell types present, not just tumour.

The relationship between MGMT and tumour sensitivity to temozolomide, if one exists, is unlikely to be straightforward: where methylated DNA is not repaired other factors, such as mismatch repair and p53 status, will influence whether the cell dies. Cell lines deficient in mismatch repair tolerate alkylation damage (Branch et al, 1993; Kat et al, 1993), and it has recently been suggested that this deficiency overrides MGMT in conferring resistance to temozolomide (Liu et al, 1996; Wedge et al, 1996).

Results should soon be available from clinical trials with $O^6$-benzylguanine, an MGMT inactivator, in combination with $O^6$-alkylating agents. The ability of this, or similar inactivators, to enhance the efficacy of alkylating agent chemotherapy will help determine the functional role that MGMT plays in tumour resistance to these therapies. Meanwhile, further work is needed in malignant melanoma to determine tumour-specific MGMT levels via immunohistochemistry, and in the investigation of alternative mechanisms of resistance to $O^6$-alkylators such as mismatch repair deficiency.

### ACKNOWLEDGEMENT

This trial was performed under the auspices of the Cancer Research Campaign Phase I/II Committee.

### REFERENCES

Bleehen N, Newlands E, Lee S, Thatcher N, Selby P, Calvert A, Rustin G, Brampton M and Stevens M (1995) Cancer Research Campaign phase II trial of temozolomide in metastatic melanoma. *J Clin Oncol* **13**: 910–913

Bower M, Newlands E, Bleehen N, Brada M, Begent R, Calvert H, Colquhoun I, Lewis P and Brampton M (1997) Multicentre CRC phase II trial of temozolomide in recurrent or progressive high-grade glioma. *Cancer Chemother Pharmacol* **40**: 484–488

Branch P, Aquilina G, Bignami M and Karran P (1993) Defective mismatch binding and a mutator phenotype in cells tolerant to DNA damage. *Nature* **362**: 652–654

Catapano C, Broggini M, Erba E, Ponti M, Mariani L, Citti L and D'Incalci M (1987) In vitro and in vivo methazolastone-induced DNA damage and repair in L-1210 leukaemia sensitive and resistant to chloroethylnitrosoureas. *Cancer Res* **47**: 4884–4889

D'Incalci M, Citti L, Taverna P and Catapano C (1988) Importance of DNA repair enzyme $O^6$-alkyltransferase (AT) in cancer chemotherapy. *Cancer Treat Rev* **15**: 279–292

Eghyazi S, Hansson J and Ringborg U (1995) $O^6$-methylguanine-DNA methyltransferase activities in biopsies of human melanoma tumours. *Br J Cancer* **71**: 37–39

Franchi A, Papa G, D'Atri S, Piccioni D, Masi M and Bonmassar E (1992) Cytotoxic effects of dacarbazine in patients with acute myelogenous leukaemia: a pilot study. *Haematologica* **77**: 146–150

Griffin S, Branch P, Xu Y and Karran P (1994) DNA mismatch binding and incision at modified guanine bases by extracts of mammalian cells: implications for tolerance to DNA methylation damage. *Biochemistry* **33**: 4787–4793

Karran P and Bignami M (1992) Self-destruction and tolerance in resistance of mammalian cells to alkylation damage. *Nucleic Acids Res* **20**: 2933–2940

Kat A, Thilly W, Fang W, Longley M, Li G and Modrich P (1993) An alkylation-tolerant mutator cell line is deficient in strand-specific mismatch repair. *Proc Natl Acad Sci USA* **90**: 6424–6428

Lee S, Thatcher N and Margison G (1991) $O^6$-alkylguanine-DNA alkyltransferase depletion and regeneration in human peripheral lymphocytes following dacarbazine and fotemustine. *Cancer Res* **51**: 619–623

Lee S, Rafferty J, Elder R, Fan C, Bromley M, Harris M, Thatcher N, Potter P, Altermatt H, Perinat-Frey T, Cerny T, O'Connor P and Margison G (1992) Immunohistological examination of the inter- and intracellular distribution of $O^6$-alkylguanine-DNA-alkyltransferase in human liver and melanoma. *Br J Cancer* **66**: 355–360

Lee S, Betticher D and Thatcher N (1995) Melanoma: chemotherapy. *Br Med Bull* **51**: 609–630

Liu L, Markowitz S and Gerson S (1996) Mismatch repair mutations override alkyltransferase in conferring resistance to temozolomide but not to 1,3-bis(2-chloroethyl)nitrosourea. *Cancer Res* **56**: 5375–5379

Major G, Gardner E and Lawley P (1991) Direct assay for $O^6$-methylguanine-DNA methyltransferase and comparison of detection methods for the methylated enzyme in polyacrylamide gels and electroblots. *Biochem J* **277**: 89–96

Margison G and O'Connor P (1990) Biological consequences of reactions with DNA: role of specific lesions. In *Handbook of Experimental Pharmacology*, Cooper C and Grover P (eds), pp. 547–571. Springer-Verlag: Berlin

Miller A, Hoogstraten B, Staquet M and Berad D (1981). Reporting results of cancer treatment. *Cancer* **47**: 207–214

Newlands E, Stevens M, Wedge S, Wheelhouse R and Brock C (1997) Temozolomide: a review of its discovery, chemical properties, preclinical development and clinical trials. *Cancer Treat Rev* **23**: 35–61

© Cancer Research Campaign 1998 

Pegg A (1990) Mammalian $O^6$-alkylguanine-DNA alkyltransferase: regulation and importance in response to alkylating carcinogenic and therapeutic agents. *Cancer Res* **50**: 6119–6129

Voigt J and Topal M (1995) $O^6$-methylguanine-induced replication blocks. *Carcinogenesis* **16**: 1775–1782

Wedge S, Porteus J and Newlands E (1996) 3-aminobenzamide and/or $O^6$-benzylguanine evaluated as an adjuvant to temozolomide or BCNU treatment in cell lines of variable mismatch repair status and $O^6$-alkylguanine-DNA alkyltransferase activity. *Br J Cancer* **74**: 1030–1036

World Health Organization (1979) *WHO Handbook for Reporting Results of Cancer Treatment*. Offset publication number 48. WHO: Geneva

Yanagisawa T, Watanabe K, Minuera K and Kowada M (1996) Measurement of $O^6$-methylguanine-DNA methyltransferase activity using oligonucleotides and restriction enzyme in human brain tumours. *Int J Oncol* **9**: 781–786

Yarosh D, Hurst-Calderone S, Babich M and Day III R (1986) Inactivation of $O^6$-methylguanine-DNA methyltransferase and sensitization of human tumour cells to killing by chloroethylnitrosourea by $O^6$-methylguanine as a free base. *Cancer Res* **46**: 1663–1668

© Cancer Research Campaign 1998


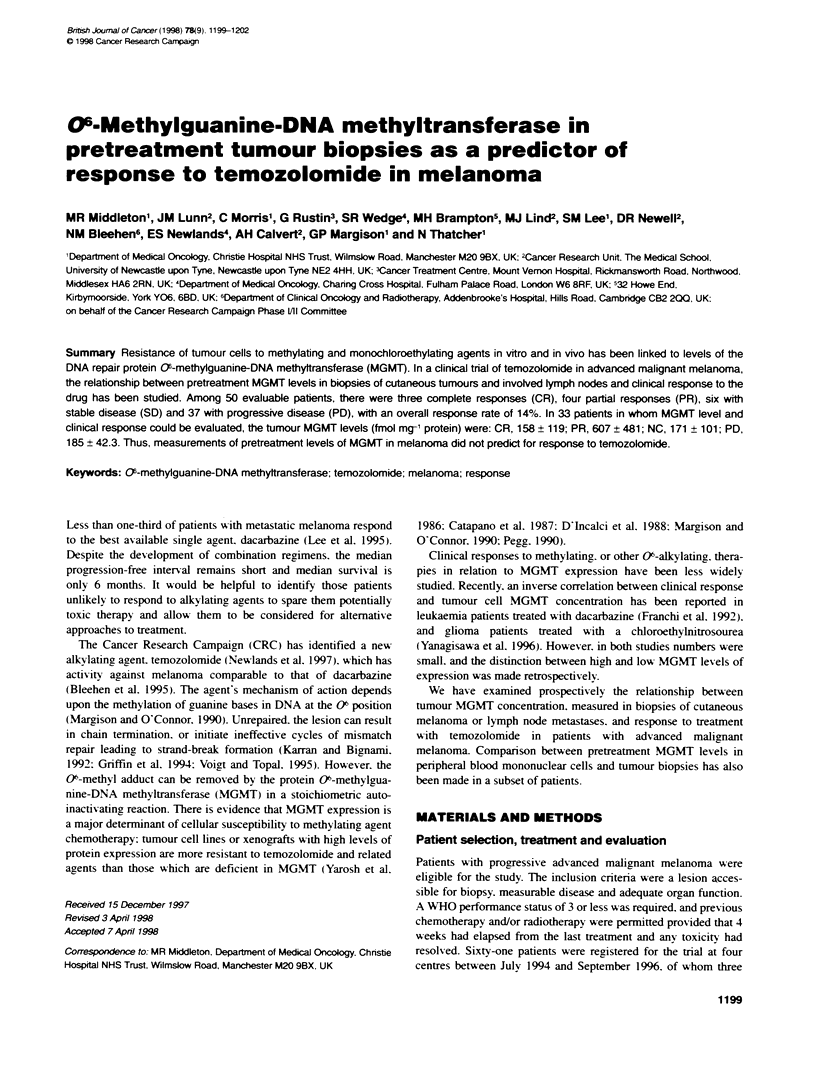

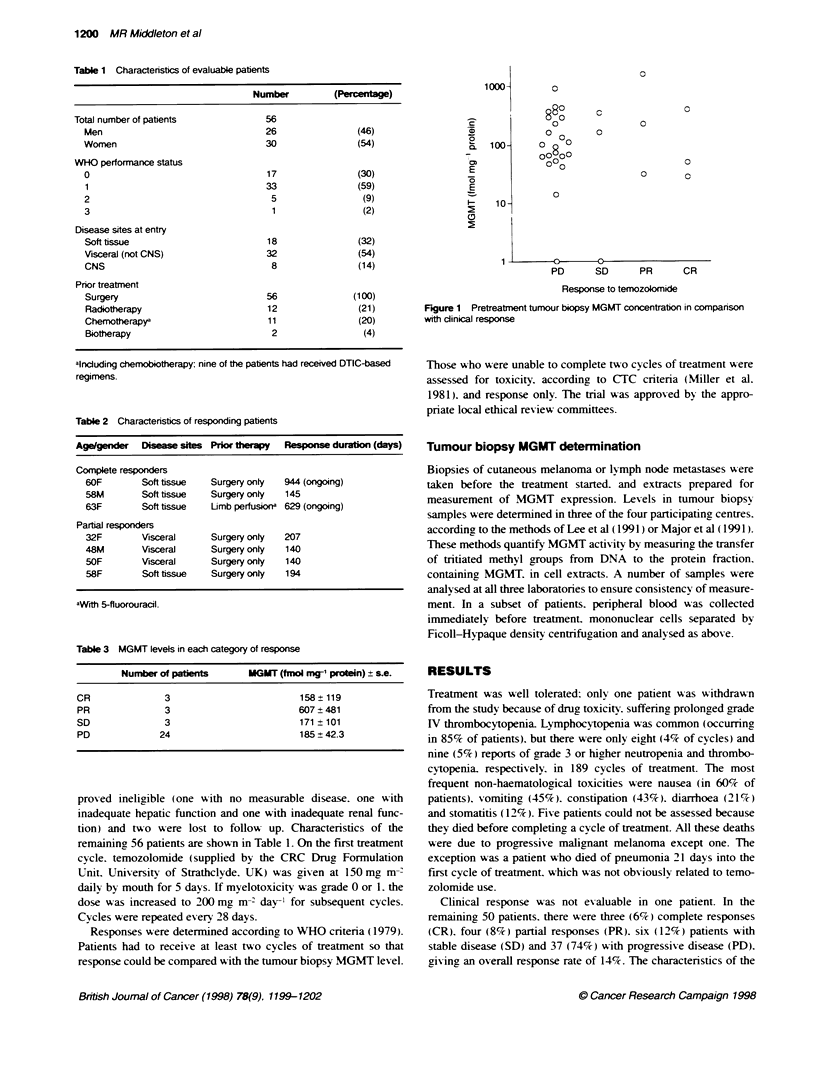

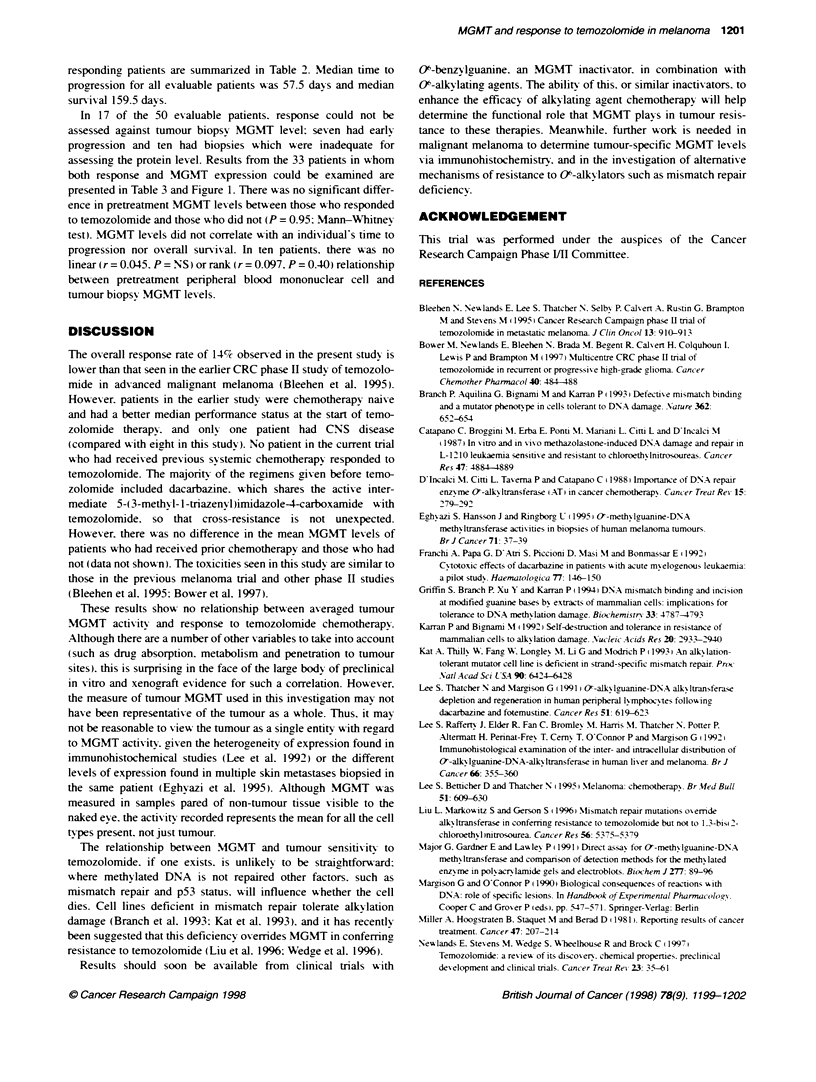

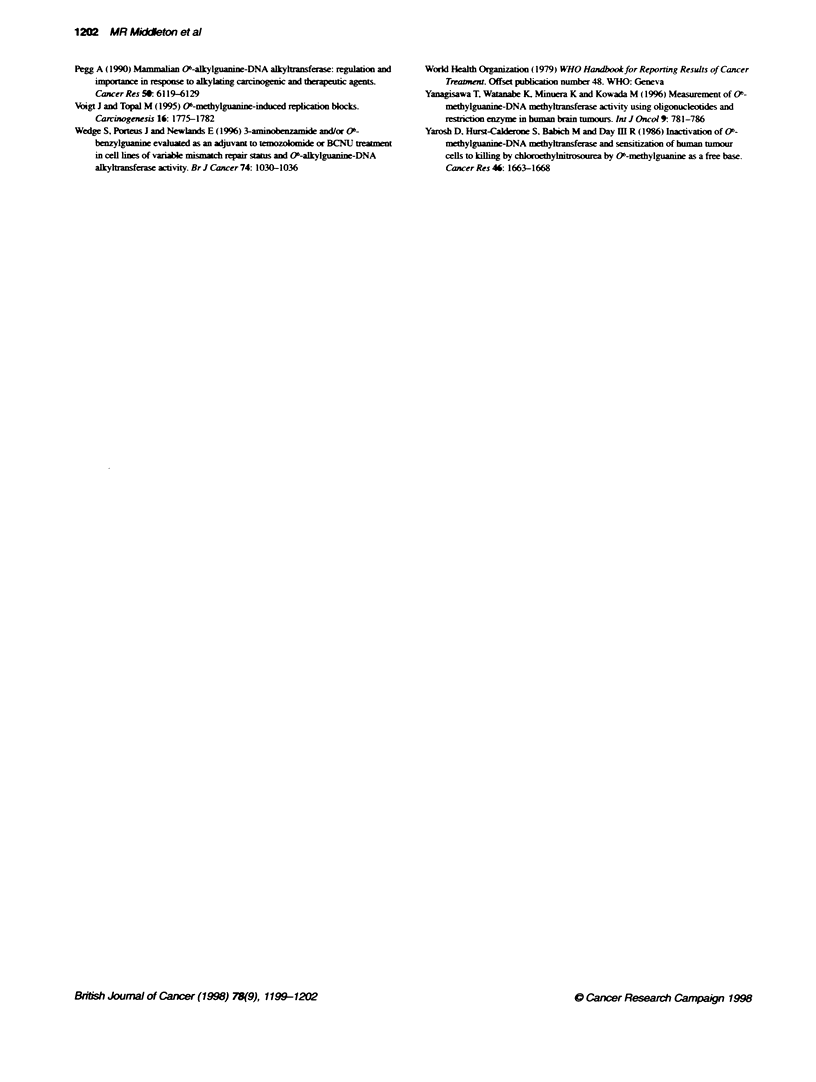


## References

[OCR_00329] Bleehen N. M., Newlands E. S., Lee S. M., Thatcher N., Selby P., Calvert A. H., Rustin G. J., Brampton M., Stevens M. F. (1995). Cancer Research Campaign phase II trial of temozolomide in metastatic melanoma.. J Clin Oncol.

[OCR_00333] Bower M., Newlands E. S., Bleehen N. M., Brada M., Begent R. J., Calvert H., Colquhoun I., Lewis P., Brampton M. H. (1997). Multicentre CRC phase II trial of temozolomide in recurrent or progressive high-grade glioma.. Cancer Chemother Pharmacol.

[OCR_00338] Branch P., Aquilina G., Bignami M., Karran P. (1993). Defective mismatch binding and a mutator phenotype in cells tolerant to DNA damage.. Nature.

[OCR_00343] Catapano C. V., Broggini M., Erba E., Ponti M., Mariani L., Citti L., D'Incalci M. (1987). In vitro and in vivo methazolastone-induced DNA damage and repair in L-1210 leukemia sensitive and resistant to chloroethylnitrosoureas.. Cancer Res.

[OCR_00351] D'Incalci M., Citti L., Taverna P., Catapano C. V. (1988). Importance of the DNA repair enzyme O6-alkyl guanine alkyltransferase (AT) in cancer chemotherapy.. Cancer Treat Rev.

[OCR_00356] Egyházi S., Hansson J., Ringborg U. (1995). O6-methylguanine-DNA methyltransferase activities in biopsies of human melanoma tumours.. Br J Cancer.

[OCR_00359] Franchi A., Papa G., D'Atri S., Piccioni D., Masi M., Bonmassar E. (1992). Cytotoxic effects of dacarbazine in patients with acute myelogenous leukemia: a pilot study.. Haematologica.

[OCR_00364] Griffin S., Branch P., Xu Y. Z., Karran P. (1994). DNA mismatch binding and incision at modified guanine bases by extracts of mammalian cells: implications for tolerance to DNA methylation damage.. Biochemistry.

[OCR_00369] Karran P., Bignami M. (1992). Self-destruction and tolerance in resistance of mammalian cells to alkylation damage.. Nucleic Acids Res.

[OCR_00375] Kat A., Thilly W. G., Fang W. H., Longley M. J., Li G. M., Modrich P. (1993). An alkylation-tolerant, mutator human cell line is deficient in strand-specific mismatch repair.. Proc Natl Acad Sci U S A.

[OCR_00391] Lee S. M., Betticher D. C., Thatcher N. (1995). Melanoma: chemotherapy.. Br Med Bull.

[OCR_00385] Lee S. M., Rafferty J. A., Elder R. H., Fan C. Y., Bromley M., Harris M., Thatcher N., Potter P. M., Altermatt H. J., Perinat-Frey T. (1992). Immunohistological examination of the inter- and intracellular distribution of O6-alkylguanine DNA-alkyltransferase in human liver and melanoma.. Br J Cancer.

[OCR_00380] Lee S. M., Thatcher N., Margison G. P. (1991). O6-alkylguanine-DNA alkyltransferase depletion and regeneration in human peripheral lymphocytes following dacarbazine and fotemustine.. Cancer Res.

[OCR_00397] Liu L., Markowitz S., Gerson S. L. (1996). Mismatch repair mutations override alkyltransferase in conferring resistance to temozolomide but not to 1,3-bis(2-chloroethyl)nitrosourea.. Cancer Res.

[OCR_00410] Miller A. B., Hoogstraten B., Staquet M., Winkler A. (1981). Reporting results of cancer treatment.. Cancer.

[OCR_00414] Newlands E. S., Stevens M. F., Wedge S. R., Wheelhouse R. T., Brock C. (1997). Temozolomide: a review of its discovery, chemical properties, pre-clinical development and clinical trials.. Cancer Treat Rev.

[OCR_00432] Wedge S. R., Porteous J. K., Newlands E. S. (1996). 3-aminobenzamide and/or O6-benzylguanine evaluated as an adjuvant to temozolomide or BCNU treatment in cell lines of variable mismatch repair status and O6-alkylguanine-DNA alkyltransferase activity.. Br J Cancer.

